# Intergroup Contact, COVID-19 News Consumption, and the Moderating Role of Digital Media Trust on Prejudice Toward Asians in the United States: Cross-Sectional Study

**DOI:** 10.2196/22767

**Published:** 2020-09-25

**Authors:** Jiun-Yi Tsai, Joe Phua, Shuya Pan, Chia-chen Yang

**Affiliations:** 1 School of Communication Northern Arizona University Flagstaff, AZ United States; 2 Grady College of Journalism and Mass Communication University of Georgia Athens, GA United States; 3 School of Journalism and Communication Renmin University of China Beijing China; 4 School of Educational Foundations, Leadership and Aviation Oklahoma State University Stillwater, OK United States

**Keywords:** COVID-19, prejudice, news exposure, news trust, infodemic, media bias, racism, social media use, intergroup contact, regression, moderation analysis, cross-sectional survey

## Abstract

**Background:**

The perceived threat of a contagious virus may lead people to be distrustful of immigrants and out-groups. Since the COVID-19 outbreak, the salient politicized discourses of blaming Chinese people for spreading the virus have fueled over 2000 reports of anti-Asian racial incidents and hate crimes in the United States.

**Objective:**

The study aims to investigate the relationships between news consumption, trust, intergroup contact, and prejudicial attitudes toward Asians and Asian Americans residing in the United States during the COVID-19 pandemic. We compare how traditional news, social media use, and biased news exposure cultivate racial attitudes, and the moderating role of media use and trust on prejudice against Asians is examined.

**Methods:**

A cross-sectional study was completed in May 2020. A total of 430 US adults (mean age 36.75, SD 11.49 years; n=258, 60% male) participated in an online survey through Amazon’s Mechanical Turk platform. Respondents answered questions related to traditional news exposure, social media use, perceived trust, and their top three news channels for staying informed about the novel coronavirus. In addition, intergroup contact and racial attitudes toward Asians were assessed. We performed hierarchical regression analyses to test the associations. Moderation effects were estimated using simple slopes testing with a 95% bootstrap confidence interval approach.

**Results:**

Participants who identified as conservatives (β=.08, *P*=.02), had a personal infection history (β=.10, *P*=.004), and interacted with Asian people frequently in their daily lives (β=.46, *P*<.001) reported more negative attitudes toward Asians after controlling for sociodemographic variables. Relying more on traditional news media (β=.08, *P*=.04) and higher levels of trust in social media (β=.13, *P*=.007) were positively associated with prejudice against Asians. In contrast, consuming news from left-leaning outlets (β=–.15, *P*=.001) and neutral outlets (β=–.13, *P*=.003) was linked to less prejudicial attitudes toward Asians. Among those who had high trust in social media, exposure had a negative relationship with prejudice. At high levels of trust in digital websites and apps, frequent use was related to less unfavorable attitudes toward Asians.

**Conclusions:**

Experiencing racial prejudice among the Asian population during a challenging pandemic can cause poor psychological outcomes and exacerbate health disparities. The results suggest that conservative ideology, personal infection history, frequency of intergroup contact, traditional news exposure, and trust in social media emerge as positive predictors of prejudice against Asians and Asian Americans, whereas people who get COVID-19 news from left-leaning and balanced outlets show less prejudice. For those who have more trust in social media and digital news, frequent use of these two sources is associated with lower levels of prejudice. Our findings highlight the need to reshape traditional news discourses and use social media and mobile news apps to develop credible messages for combating racial prejudice against Asians.

## Introduction

### Background

Since the COVID-19 outbreak, the United States has become an epicenter, surpassing 5 million confirmed cases as of August 2020. The behavioral immune system theory posits that people’s tendency to avoid the risk of pathogen contagion is driven by cognitive and affective responses to informational cues [1-3]. Evidence supports that the perceived threat of a contagious virus will provoke strong aversive emotions, leading people to be distrustful of immigrants and out-groups [4,5]. Indeed, reports of anti-Asian racial incidents and hate crimes in the United States reached over 2000 since the term “Chinse virus” was used [6]. A national survey collected in March 2020 found 42% of US residents were likely to engage in discriminatory behaviors toward Asians because of their fear of the virus [7]. Relatedly, the Pew Research Center documented that 40% of Americans believed racial bias against Asians was more common than it was before the outbreak, and 31% of Asian Americans have experienced slurs or racist jokes since the pandemic [8].

Two psychological mechanisms are relevant in explaining the sharp increase in prejudice against out-groups when people cope with high levels of anxiety about infectious diseases. First, the contact hypothesis [9-11] postulates that positive face-to-face intergroup contact will improve out-group attitudes, contributing to prejudice reduction. Conversely, negative intergroup contact could elicit hostility toward out-groups. Because COVID-19 is transmitted through interpersonal interaction, the salience and magnitude of a perceived threat may result in either avoidance of Asians who are historically associated with diseases or hostile attitudes toward Asians [12].

Second, consuming information in news media and social media offers indirect forms of mediated or vicarious contact, thus shaping attitudes toward out-groups when people have minimal direct interaction with minority groups during shelter-in-place orders [13,14]. Early media coverage of the COVID-19 pandemic disproportionately focused on Chinese citizens’ consumption of raw bats and other wild animals [15]. Particularly on the internet, conspiracy theories and racist scapegoating of China arose, with widely shared articles, social media posts, and viral videos blaming Chinese people, and Asians in general, for their “dirty” and “unsanitary” eating habits [16]. At the same time, the US government officials’ politically consequential messages such as referring to the virus as Chinese virus were prevalent in national press briefings [17]. The constant coverage in mainstream news and sharing on social media likely heightened the salience of infection risk and the impression of Chinese and Asian people as a threat to health in the public’s mind. As a majority of Americans have been closely following COVID-19 news from legacy media, local news, and social media [18-21], the prevalent rhetoric blaming China and the negative racial stereotypes can play an influential role in activating prejudice against Asians [22,23].

To date, little research has linked the two aforementioned mechanisms to bias toward stigmatized groups in the context of a global pandemic. Based on a cross-sectional online survey of 430 participants, we jointly examined two psychological mechanisms underlying the activation of prejudice against Asians and Asian Americans in the United States during the COVID-19 pandemic: intergroup contact and indirect contact of media consumption. To reflect the hyper-choice and hyper-partisan news environment, we differentiated three functionally different concepts of media consumption: media use, media trust, and media bias. Next, we compared how traditional news, social media use, and news apps cultivated racial attitudes. Last, this research lent empirical support to the moderating role of media use and trust in prejudice against Asians. Together, the timeliness of our results will yield theoretical and practical insights into developing public health interventions for reducing racial discrimination linked to the COVID-19 pandemic.

### Intergroup Contact and Prejudice

Literature on the behavioral immune system has documented that humans’ subjective risk perceptions of contagious diseases will trigger a series of cognitive, affective, and behavior mechanisms to avoid pathogen infection [1,2]. The tendency of pathogen avoidance is integral to coping with infectious diseases. The salience of disease threats is often activated by situational cues such as media reports of virus outbreaks and disgusting images, thereby triggering pathogen-avoidant cognitions and behavioral avoidance [3]. Empirical evidence shows that salient disease cues increase the tendency to avoid people who are potentially infectious [24]. Moreover, the activation of the behavior immune activity could motivate aversion and prejudicial attitudes toward out-groups, especially minority members historically associated with dirtiness and diseases [24]. In sum, disease threats can sensitize people to risks of intergroup contact with visibly stigmatized members.

Despite a lower fatality rate than Middle East respiratory syndrome (MERS) and Ebola, the SARS-CoV-2 virus has caused one of the highest death tolls worldwide, and there is still no vaccine available [25]. As the surges of confirmed COVID-19 cases continue to dominate US media attention, the salience and magnitude of the perceived threat is heightened. The sensitivity of intergroup risks activated by constant rhetorical cues of blaming Chinese people can possibly explain an increase in prejudice against Asians. Through three online experiments, Huang et al [4] found that participants felt more negative about immigrants after reading a news report about the 2009 swine flu health risks and shortage of vaccine supply. Kim et al [26] conducted a national sample survey of 1000 American adults during the 2014 Ebola outbreak. Findings concluded that vulnerable people had more generalized xenophobia. Given that emotional attitudes toward out-groups are more closely related to subsequent discriminatory behaviors, we focus on affective feelings about Asians as a proxy for measuring prejudice [27].

### Role of Media Use, Trust, and Source Bias in Out-Group Attitudes

In addition to serving as situational cues to activate aversion of minority groups, news reports offer indirect mediated contact that possibly shapes prejudice toward ethnic minorities [13,14]. Different from prior research, we differentiate three functionally different concepts of news consumption: modalities of media use, media trust, and media bias. We further offer an overview of how each concept relates to negative attitudes toward out-groups.

Decades of cultivation theory and news framing research have proved the direct influence of using traditional media, mostly newspapers and TV newscasts, on cultivating audiences’ prejudicial views of out-groups [28]. Persistent stereotypical media coverage of a particular racial group would result in prejudicial beliefs toward individuals of such group. For instance, Dixon [29] found that network TV news exposure resulted in more stereotypical views and negative perceptions of African Americans. Another study [30] examined racialized news framing of President Barack Obama, suggesting that exposure to negative frames about Obama activated underlying prejudicial beliefs and biased evaluations of African Americans in general. Likewise, negative TV portrayals of out-groups such as refugees increased prejudicial attitudes toward refugees based on experimental designs [14]. Therefore, the traditional news coverage of politicized COVID-19 discourses placing blame on China, along with the labeling of the disease as a “Chinese virus” or a “Wuhan virus,” might have a negative impact on racial perceptions and stereotypes of Asian Americans and Asians in general, leading to prejudice against them [22,23].

Although the aforementioned studies are insightful, relying on one average scale to measure individuals’ exposure to traditional media outlets (eg, print newspaper and TV) limits our understanding of how American audiences receive information from hyper-choice, hyper-partisan, networked news environments [13,14,23,29]. The average index approach assumes that modalities of news media consumption associate out-group attitudes equally. However, it is conceivable that different information channels emphasize distinct coverage of the pandemic and differ in the prevalence of anti-Asian discourses. Moreover, it remains unclear how the use of social media and digital outlets might be related to differential attitudes toward out-groups.

In addition to the well-documented influence of traditional media exposure on prejudice, the role of social media and digital outlets in shaping users’ views of ethnic minorities cannot be overlooked. Social media was and is an essential source for coping with the MERS outbreak and the ongoing pandemic [31-33]. At the same time, political leaders’ antiminority sentiment tended to circulate widely on social media platforms, contributing to an increase of hate crimes toward minority groups such as Muslims [34]. Although social media provides real-time updates and personally relevant feeds that might reinforce audience’s pre-existing biases, users are more likely to be susceptible to misinformation and racially offensive comments [16,20]. Analysis of 2.8 million COVID-19–related English tweets from February and March 2020 revealed that one of the top topics involved racist attacks and rude comments against East Asians [21]. Budhwani and Sun [35] found that tweets containing “Chinese virus” or “China virus” increased ten-fold after political leaders used the insensitive terms on Twitter. However, findings on linking social media use with attitudes toward stereotyped groups remain inconclusive. One study surveying US college students revealed no significant relationship between getting news from social media and prejudice toward undocumented immigrants [36]. In contrast, one recent online survey showed that believing in social media news was positively associated with the perceptions that American identity and the economic situation were threatened by the presence of Chinese people [37]. To address this gap, we will examine how stereotypes propagated on social media and online news may associate with prejudice toward Asians.

Media trust and media use are correlated but conceptually different [38]. A worldwide survey showed that traditional news exposure correlated positively with trust in TV and newspapers, whereas online news use was negatively associated with trust in traditional media [39]. Put differently, when people cope with the evolving situation of COVID-19, not all media types will be trusted equally and have a universal association with activating perceptions of racial stereotypes and prejudicial attitudes. In an increasingly fragmented and polarized media environment, we argue that media use and trust in each information source should be measured separately to generate valid conclusions about their distinct relationships with prejudice. To our knowledge, scant literature has investigated media use and trust simultaneously, nor have they probed the moderation role of viewers’ trust in each medium in the exposure-prejudice relationship. Since the outbreak, the use of TV news, online media, and social media sources grew substantially. At the same time, US public trust in news has hit a new low point. Only 29% of American showed trust in news overall in 2020, a significant decrease of 9% compared to 2017, and 14% of them trusted news from social media [40]. The public might trust legacy media more than social media because of journalists’ gatekeeping function and reporting accuracy [17,19,20]. Considering the polarization in US newspaper and TV network coverage of COVID-19 [41] and the partisan nature of news trust [42], exploring the moderating role of media trust in determining the direction of the influence of media use on prejudicial attitudes is vital.

Last, relying on conservative or liberal media could also be a significant determinant of negative attitudes toward out-groups. An online survey with 422 Italians demonstrated a positive relationship between exposure to right wing newspapers and TV newscasts, and prejudice against immigrants after controlling for participants’ political ideology. Conversely, using left-leaning news outlets showed a negative association with prejudice [43].

Guided by the behavioral immune system theory [1-5], associated outcomes of prejudice toward out-group members [24-26], and the intergroup contact hypothesis (H) [9-12], we expect that salient perceived risks of contracting COVID-19 when interacting with Asians will positively be related to prejudice.

- H1: The frequency of direct intergroup contact will be positively associated with prejudicial attitudes toward Asians.

As no prior research distinguishes how modalities of news, news trust, and media bias relate to virus-activated prejudice, we propose the following research questions (RQs):

- RQ1: How will media use, media trust, and media bias be associated with prejudicial attitudes toward Asians?
- RQ2: How will media trust moderate the relationship between media use and prejudicial attitudes toward Asians?

## Methods

### Participants and Procedure

In May 2020, we recruited participants from Amazon’s Mechanical Turk (Mturk) program, a dominant internet-based platform for gathering online samples in behavioral science fields [44]. A short description containing the research title, keywords, and expected completion time was generated in a coauthor’s Mturk account. Interested participants clicked on an external link that rerouted them to take the Qualtrics survey. Upon completion, the Mturk program generated a random code for participants to receive compensation. The Mturk subject pools demonstrate proven advantages of being more attentive than collegiate populations [44]. Although the representativeness of Mturk samples raise some concerns, Levay et al [45] concluded that Mturk respondents’ attitudes toward social issues did not differ fundamentally from the random sample of the American National Election Studies after controlling for key demographics. Similarly, Mturk samples can generate data quality comparable to representative samples [46]. Moreover, Mturk’s recruitment speed is excellent for study designs that depend on current social events [47]. For instance, Park et al [48] used Mturk samples to study American adults’ information channel preferences during the 2016 Zika virus outbreak. Given that confirmed cases in the United States has surged since March 2020, Mturk samples are suitable for our objectives because of the ability to collect how individuals relied on legacy news and social media to cope with the global pandemic in a naturalistic setting.

To determine the required sample size to achieve desired statistical power, we performed an a priori estimate using G*Power version 3.1 (Heinrich-Heine-Universität Düsseldorf) before data collection [49]. A medium regression effect size (*R*^2^=0.13) with 99% power and 18 predictors indicated a minimum sample of 275 [50]. To ensure the quality of study results, we limited the sample to people residing in the United States. Only participants who passed attention check questions were included in the analysis, yielding a final sample of 430 (mean age 36.75, SD 11.49 years; n=258, 60% male). The majority of participants were White (n=344, 80%), married (n=313, 72.8%), employed full-time (n=318, 74%), and had received Bachelor’s degrees (n=267, 62.1%), and 11.4% (n=49) reported having been infected by COVID-19. More than half of the participants (n=224, 52.1%) indicated their family income has been impacted by the outbreak. Table 1 summarizes demographic characteristics of the sample.

**Table 1:** Demographics of survey participants (N=430).

Variables		Participants
Age (years), mean (SD)		36.75 (11.49)
Male, n (%)		258 (60.0)
**Education, n (%)**		
	High school or less	15 (3.5)
	Some college	50 (11.6)
	Bachelor’s degree	267 (62.1)
	Postgraduate	98 (22.8)
**Race^a^, n (%)**		
	White	344 (80.0)
	Black or African American	52 (12.1)
	Hispanic and Latino	34 (7.9)
	Other	9 (2.1)
**Marital status, n (%)**		
	Married or domestic partnership	313 (72.8)
	Single	100 (23.3)
	Other	17 (3.9)
**Employment, n (%)**		
	Employed, ≥40 hours per week	318 (74)
	Employed, <40 hours per week	77 (17.9)
	Other	35 (8.1)
Family income impacted, n (%)		224 (52.1)
Political ideology, mean (SD)		4.08 (2.13)
Personal infection of COVID-19, n (%)		49 (11.4)

^a^Participants could select one or more self-identified races.

The Institutional Review Board office of Renmin University of China approved the research protocol, and responses were collected via Qualtrics software (Qualtrics International Inc). The first author at a US university in the southwest was provided with an anonymized data set containing no identifiable private information connected to participants. Ethics exemptions were obtained at coauthors’ institutions before launching the survey. At the beginning of the survey, the respondents agreed to participate in the research voluntarily for receiving compensation. Next, respondents answered questions related to traditional news exposure, social media use, perceived trust, and their top three news channels for staying informed about the novel coronavirus. In addition, the frequency of intergroup contact and racial attitudes toward Asians were assessed. The survey took approximately 16 minutes to complete (mean 15.95, SD 39.34 minutes).

### Measures

#### Intergroup Contact

We used a common measure [13,37,51] to assess the frequency of direct intergroup contact on a 7-point scale: “How much contact do you have with Asian people (a) at work; (b) as neighbors; (c) as close friends?” (1=none at all, 7=a great deal; Cronbach α=.88, mean 4.11, SD 1.63).

#### Media Use

Respondents rated how often they get news about COVID-19 from five traditional sources including print newspaper or magazine, radio, local TV, national network television, and cable TV on a 4-point scale with endpoints labeled “never” and “often” (Cronbach α=.72, mean 2.80, SD 0.64). Two single 4-point scales measured social media use (mean 3.12, SD 0.88) and news websites or app use (mean 3.10, SD 0.83).

#### Media Trust

To gauge different levels of media trust in news sources, we asked participants to indicate how much they trust information about COVID-19 from the aforementioned media outlets (1=not at all, 7=very much) [38]. Trust in traditional media demonstrated good internal consistency (Cronbach α=.86, mean 4.83, SD 1.20). Two single items were measured: social media trust (mean 4.72, SD 1.67) and trust in websites or apps (mean 5.20, SD 1.26).

#### Media Bias

Exposure to biased news sources was assessed by asking respondents to select the top three news channels they frequently visited for gathering COVID-19 news. We obtained a list of 31 popular news brands across a wide range of platforms from the Pew Research Center’s survey of US media polarization and the 2020 election [52]. We then determined the political leanings of each news channel based on ratings provided by AllSides [53]. AllSides employs multiple methods to identify comprehensive media bias ratings for 600 media outlets. Next, we used a 5-point scale to represent each source’s ideological placement of left (-2), lean left (-1), center (0), lean right (+1) and right (+2). Exposure to left-leaning sources was calculated by taking the absolute value of the sum of selected channels’ bias ratings (mean 1.62, SD 1.05, range 0-5). Exposure to centrist news was calculated by the raw counts that neutral or balanced outlets were selected by each participant (mean 0.73, SD 0.71, range 0-3). Lastly, exposure to right-leaning media was measured by adding up selected sources’ bias scores (mean 0.70, SD 0.87, range 0-4)

#### Prejudicial Attitudes

We modified a measure from [54] to assess prejudicial attitudes toward Asians. The question was rephrased as “Since the COVID-19 outbreak, how much have you felt the following toward Asian people in general?” Respondents indicated, from 1 “not at all” to 7 “very much,” seven negative feelings: (1) jealousy, (2) anger, (3) resentment, (4) discomfort, (5) hatred, (6) despise, and (7) shame. These items formed a reliable scale with higher values representing higher levels of prejudice toward Asians (Cronbach α=.97, mean 3.61, SD 1.83). The phrasing of “since the COVID-19 outbreak” was included to set a standard time frame as a basis for participants to respond to news consumption and disease-linked attitudes. Using specific and concrete wording is a recommended practice for survey designs [55]. Specifying a consistent time frame allowed us to pinpoint the relationships between people’s exposure and trust in COVID-19 news and negative feelings toward Asians during the pandemic.

#### Controls

As news consumption is associated with sociodemographic variables, we measured common characteristics for statistical control [13,37,43]. Respondents reported demographic characteristics including age, gender (1=male, 0=female), educational attainment, ethnicity (1=White, 0=others), marital status (1=married or domestic partnership, 0=others), and employment status (1=full-time, 0=others). Personal infection of COVID-19 was measured with one dichotomous item (1=yes, 0=no). Lastly, we measured political ideology on a 7-point scale from 1 “very liberal” to 7 “very conservative” (mean 4.08, SD 2.13).

### Statistical Analysis

Table 2 summarizes bivariate correlations of continuous variables. Next, we performed four-step hierarchical linear regression analyses using SPSS version 26 (IBM Corp) to predict prejudicial attitudes toward Asians since the COVID-19 spread. All variance inflation factor scores among predictor variables were less than 3, indicating there was no multicollinearity. The first models included respondents’ demographic characteristics and history of personal infection as controls. In the second model, we investigated the main effect of direct intergroup contact to test H1. To answer RQ1, media use, trust in each channel, and exposure to news sources with political leanings were entered in the third model. To explore RQ2, we used mean-centered media use and mean-centered trust scores to compute three separate interaction terms, which were then included in the fourth model. Interaction terms were created by multiplying use of a given medium by trust in the given medium. Subsequently, we conducted significant tests of simple slopes with a 95% bootstrap confidence interval approach in Mplus version 8 (Muthén & Muthén). Moderation effects were tested by creating 5000 bootstrap samples. Such an approach is recommended because it leads to less-inflated type I error rates and provides coverage of confidence intervals [56,57].

**Table 2:** Bivariate correlations with *P* values.

Variables			1		2		3		4		5		6		7		8		9		10		11
**1. Intergroup contact**																							
	*r*	1		0.44		0.11		0.06		0.42		0.48		0.31		–0.12		0.03		–0.01		0.64	
	*P* value	—^a^		<.001		.03		.24		<.001		<.001		<.001		.01		.60		.84		<.001	
**2. Traditional media use**																							
	*r*	0.44		1		0.38		0.21		0.66		0.50		0.40		–0.03		0.01		0.04		0.34	
	*P* value	<.001		—		<.001		<.001		<.001		<.001		<.001		.61		.86		.46		<.001	
**3. Social media use**																							
	*r*	0.11		0.38		1		0.16		0.25		0.45		0.20		0.08		–0.09		–0.06		0.07	
	*P* value	.03		<.001		—		.001		<.001		<.001		<.001		.10		.06		.24		.16	
**4. Websites or apps use**																							
	*r*	0.06		0.21		0.16		1		0.21		0.07		0.30		0.10		–0.02		0.02		–0.04	
	*P* value	.24		<.001		.001		—		<.001		<.001		<.001		.048		.72		.72		.40	
**5. Traditional media trust**																							
	*r*	0.42		0.66		0.25		0.21		1		0.51		0.56		0.03		0.05		–0.13		0.31	
	*P* value	<.001		<.001		<.001		<.001		—		<.001		<.001		.53		.28		.008		<.001	
**6. Social media trust**																							
	*r*	0.48		0.50		0.45		0.07		0.51		1		0.44		–0.06		–0.03		–0.02		0.43	
	*P* value	<.001		<.001		<.001		<.001		<.001		—		<.001		.23		.53		.74		<.001	
**7. Websites or app trust**																							
	*r*	0.31		0.40		0.20		0.30		0.56		0.44		1		0.05		0.03		–0.09		0.21	
	*P* value	<.001		<.001		<.001		<.001		<.001		<.001		—		.29		.54		.06		<.001	
**8. Left-leaning**																							
	*r*	–0.12		–0.03		0.08		0.10		0.03		–0.06		0.05		1		–0.45		–0.37		–0.18	
	*P* value	.01		.61		.10		.048		.53		.23		.29		—		<.001		<.001		<.001	
**9. Centrist**																							
	*r*	0.03		0.01		–0.09		–0.02		0.05		–0.03		0.03		–0.45		1		–0.21		–0.07	
	*P* value	.60		.86		.06		.72		.28		.53		.54		<.001		—		<.001		.15	
**10. Right-leaning**																							
	*r*	–0.01		0.04		–0.06		0.02		–0.13		–0.02		–0.09		–0.37		–0.21		1		0.05	
	*P* value	.84		.46		.24		.72		.008		.74		.06		<.001		<.001		—		.30	
**11. Prejudice**																							
	*r*	0.64		0.34		0.07		–0.04		0.31		0.43		0.21		–0.18		–0.07		0.05		1	
	*P* value	<.001		<.001		.16		.40		<.001		<.001		<.001		<.001		.15		.30		—	

^a^Not applicable.

## Results

Table 3 presents the results of the hierarchical regression models predicting prejudicial attitudes toward Asians. Demographic variables and COVID-19 infection history significantly explained 25% of the variance in prejudice (*F*_8,421_=17.65, *P*<.001). Specifically, participants who were married (β=.13, *P*=.001), held conservative beliefs (β=.08, *P*=.02), and had a personal infection (β=.10, *P*=.004) reported more negative attitudes toward Asians.

**Table 3:** Hierarchical regression analysis predicting prejudicial attitudes toward Asians since COVID-19 (N=430).

Variables		Model 1^a^, β	*P* value	Model 2, β	*P* value	Model 3^b^, β	*P* value	Model 4^b^, β	*P* value
Age		–.15	.001	–.06	.11	–.05	.18	–.02	.54
Male		.01	.87	–.06	.11	–.06	.10	–.05	.19
Education		.15	.001	.04	.29	.04	.29	.02	.60
White		–.06	.14	–.02	.62	–.03	.46	–.03	.40
Married		.27	<.001	.18	<.001	.14	<.001	.13	.001
Employed full-time		.06	.15	.01	.73	–.01	.83	–.01	.90
Political ideology		.14	.001	.09	.01	.08	.04	.08	.02
Personal infection		.23	<.001	.13	<.001	.12	.001	.10	.004
Intergroup contact		N/A^c^	N/A	.55	<.001	.47	<.001	.46	<.001
**Media use**									
	Traditional media	N/A	N/A	N/A	N/A	.09	.04	.08	.04
	Social media	N/A	N/A	N/A	N/A	–.05	.16	–.06	.06
	Websites or apps	N/A	N/A	N/A	N/A	–.05	.16	–.06	.13
**Media trust**									
	Traditional media	N/A	N/A	N/A	N/A	.03	.59	.05	.36
	Social media	N/A	N/A	N/A	N/A	.14	.005	.13	.007
	Websites or apps	N/A	N/A	N/A	N/A	–.02	.60	–.05	.26
**Media sources**									
	Left-leaning	N/A	N/A	N/A	N/A	–.16	.001	–.15	.001
	Centrist	N/A	N/A	N/A	N/A	–.14	.003	–.13	.003
	Right-leaning	N/A	N/A	N/A	N/A	–.04	.35	–.03	.52
**Interaction**									
	Traditional media use * trust	N/A	N/A	N/A	N/A	N/A	N/A	–.02	.57
	Social media use * trust	N/A	N/A	N/A	N/A	N/A	N/A	–.12	.003
	Websites/apps use * trust	N/A	N/A	N/A	N/A	N/A	N/A	–.13	<.001
*R* ^2^		0.25	<.001	0.49	<.001	0.53	<.001	0.56	<.001
*R*^2^ change		N/A	N/A	0.24	<.001	0.04	<.001	0.03	<.001

^a^Standardized beta coefficients (β) are reported.

^b^In models 3 and 4, scores of media use and trust are mean-centered.

^c^N/A: not applicable.

Turning to the first hypothesis, we found a positive association between intergroup contact and prejudice (β=.46, *P*<.001) after controlling for demographic characteristics, supporting H1. Respondents who interacted with Asian people frequently in their daily lives were more inclined to hold unfavorable attitudes toward Asians.

For RQ1, media use, trust, and reliance on biased sources showed significantly divergent relationships with prejudicial attitudes toward Asians (*R^2^* change 0.04, *F*_18,411_=25.23, *P*<.001). Reading print newspapers and watching TV newscasts about COVID-19 was positively associated with higher levels of prejudice (β=.08, *P*=.04). Regarding the main effects of media trust, the results suggested that the more people trusted news circulated on social media, the higher their prejudicial attitudes were (β=.13, *P*=.007). Trust in traditional media and digital channels had no significant associations with negative feelings about Asians. Notably, relying on liberal media (β=–.15, *P*=.001) and balanced news sources (β=–.13, *P*=.003) for staying informed about the COVID-19 epidemic was significantly associated with lower levels of negative attitudes toward Asians.

**Figure 1:**
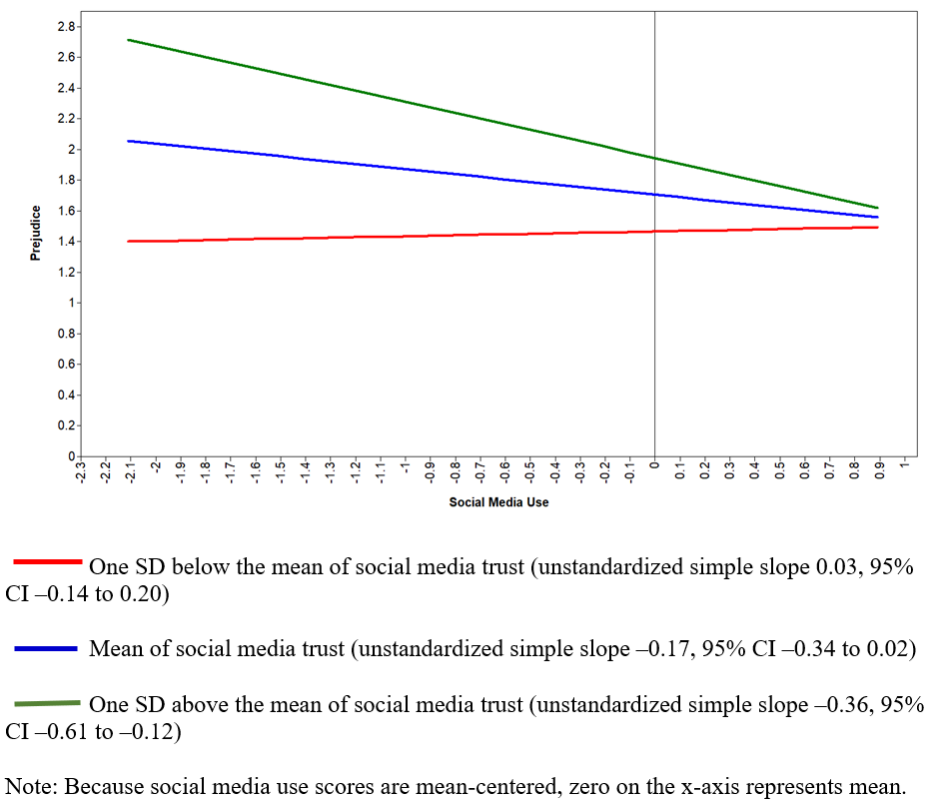
Interaction plot showing predicted values of prejudicial attitudes toward Asians as a function of social media use and trust in social media.

**Figure 2:**
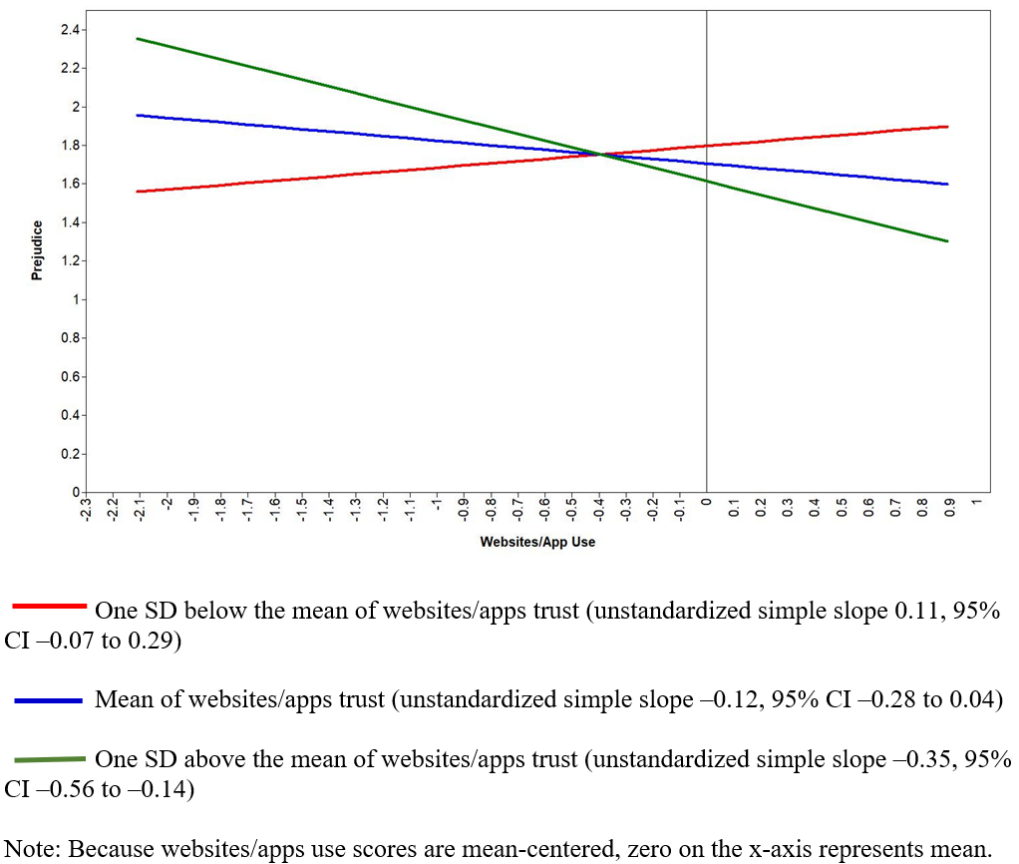
Interaction plot showing predicted values of prejudicial attitudes toward Asians as a function of websites/apps use and trust in websites/apps.

The second RQ explored whether news trust moderated the relationship between media use and prejudice against Asians. Interaction terms explained a 3% increase in variance in prejudice (*R*^2^ change 0.03, *F*_21,408_=24.33, *P*<.001). The interaction term of trust and social media use was statistically significant (β=–.12, *P*=.003). We also found a similar significant interaction effect of websites and apps trust and use on prejudice (β=–.13, *P*<.001). To explore how variation in media trust changes the relationship between use of social media or digital news and prejudice, we conducted simple slopes analyses. As depicted in Figure 1, the negative relationship between social media use and prejudicial attitudes was significantly greater among participants who had higher levels of trust in social media news, when compared to those with medium and low trust in social media (unstandardized simple slope –0.36, 95% CI –0.61 to –0.12). Additionally, Figure 2 illustrates that, at high levels of trust in online websites and mobile apps, frequent use of digital media was related to less unfavorable attitudes toward Asians (unstandardized simple slope for high trust –0.35, 95% CI –0.56 to –0.14).

## Discussion

### Principal Findings

Drawing from the behavioral immune system theory, intergroup relations, and media psychology literature, this study is a novel attempt to investigate the joint mechanisms of direct intergroup contact and mediated contact offered by diverse news sources on associating with prejudicial attitudes toward Asians during the COVID-19 outbreak. In accordance with previous studies [1-5,7,9-13,24,26], participants who subscribed to conservative beliefs, had personal COVID-19 infection history, and interacted with Asian people frequently in their daily lives reported more negative attitudes toward Asians after controlling for sociodemographic variables. It might be plausible that people with conservative beliefs are more wary of China’s influence, making them more likely to blame visible targets with Asian-looking features during the pandemic. Importantly, the medium effect size between intergroup contact and prejudice underscores the role of direct social interaction in relating to people’s out-group attitudes triggered by pandemic threats. As expected, human tendency to avoid contagious diseases will stimulate aversive emotions when interacting with minorities explicitly associated with spreading COVID-19. Frequent contact with stigmatized Asian members during the pandemic might increase perceived risks of contracting the virus and, thus, link to negative attitudes.

We empirically examined the associations between three forms of indirect mediated contact and prejudice. The findings reveal the divergent role of news exposure, trust, and political-leaning media in racial attitudes when political orientation and intergroup contact are controlled for. Relying more on traditional news media was positively related to prejudice toward Asians, supporting the TV news’ capability to activate or reinforce prevalent racial stereotypes [28-30]. In line with a current report [40], the survey participants watched more national, local, and cable television news rather than receiving information from print media. The positive association can be mostly driven by the fact that TV newscasts live streaming the President’s daily press briefings increased viewers’ firsthand exposure to the China-blaming discourses. Participants tend to hold unfavorable attitudes toward negatively portrayed groups on TV because repeated exposure to such politicized messages makes accessible the audiences’ cognitive schemas of associating Asians or Chinese immigrants with disease threats during an unfamiliar pandemic [41,58].

Regression results suggest habitual selections of news outlets with ideological leanings are a more driven factor than the modalities of media in associating with disease-activated racial attitudes. Consuming news from left-leaning and neutral outlets was associated with less prejudicial attitudes toward Asians holding other factors constant. Journalistic powerhouses with liberal and centrist perspectives are more critical of the current administration’s effort in curbing the pandemic and attribute the blame to uncoordinated policy responses at all levels of governments. Although conservative news such as Fox and liberal outlets such as CNN shared similar keyword use of mentioning China and the novel virus, a recent study found CNN news stories featured more scientific statements from Dr Anthony Fauci and Dr Deborah Birx, and emphasized more protective responses such as social distancing and lockdowns than their conservative counterparts did [59]. Liberal and centrist media’s emphasis on scientific evidence and prevention strategies possibly explains why those relying on these sources for COVID-19 news hold less prejudice and perceived threats by the presence of Asians.

It is worth highlighting the positive link between social media trust and prejudice. Trust in social media was positively associated with prejudicial attitudes toward Asians. The nature of filter algorithms and selective exposure patterns might afford social media users to be exposed to or spread unfiltered discriminating language about Asians. Using social media as news sources increases users’ probability to encounter trending anti-Asian sentiment, even when users do not actively seek such information. People tend to trust information created and shared by friends and family members within homogenous networks [60]. Recent studies [21,35,61] found that hate speech and racist attacks against Asians sharply increased on Twitter after the World Health Organization recognized COVID-19 as a global pandemic in mid-March. Common tweet keywords associated with China were eating habits, animals, virus, blame, and cause, echoing the China-blaming discourses [35,61]. Therefore, the more people trust social media (and thus the stigmatizing comments in this channel), the more likely they would perceive Asian people as the major responsible party for spreading the virus, thereby triggering negative attitudes.

Notably, this study contributes to existing literature by identifying trust in social media and digital websites and apps as a boundary condition of the news exposure-prejudice relationship. Among those who used social media infrequently, those who trusted social media more reported higher prejudice against Asians than those who trusted social media less. However, this difference became less pronounced among heavy users. Likewise, for those who had high levels of trust in digital websites and apps, frequent use was related to less unfavorable attitudes toward Asians. It could be possible that heavy users of social media and digital news have advanced media literacy of filter algorithms, equipping them with higher efficacy to move beyond personalized feeds and search for less politicized information and diverse perspectives. In other words, frequent use of social media and digital sources buffers the relationship between trust in social media and digital apps, and prejudice linked to COVID-19. Together, these results suggest that researchers should consider using nuanced measures of media exposure, media trust, and media bias to generate more accurate conclusions. In a hyper-choice networked news environment, Americans’ use of and trust in traditional media, social media, and well-known news brands with distinct political leanings have more complex relationships with their disease-ignited prejudice toward minority groups than previously assumed.

### Limitations and Future Directions

We acknowledge limitations of this study. First, the self-selected sample might reflect respondents’ heightened concerns about COVID-19–related topics. In addition, the cross-sectional study did not guarantee the causal effects of intergroup contact and news use on influencing racial attitudes. Hence, it is possible that participants who held negative views about the Chinese government or Chinese people selected to consume anti-Asian coverage on traditional outlets and social media because those news reports confirmed their existing viewpoints. Given our correlational findings, longitudinal studies will expand upon the results to establish the causal relationships. Second, the study did not directly measure the prevalence of anti-Asian discourses circulated via all media outlets since the major outbreak in the United States. However, a recent study analyzing Google Trends data between December 2019 to March 2020 showed that the “Chinese virus” rhetoric has led to a sharp increase in search rates for anti-Chinese slurs. Therefore, we can confidently infer that an increase in search rates reflected trending news reports and social media discussion related to anti-Asian sentiment and racist attacks [16].

First, in light of these limitations, future research should content analyze the prevalence of anti-Asian sentiment on different types of media and correlate with public attitudes to replicate these findings. Second, as citizens can access the same news content from multiple modalities, conventional categorizations of media use might not capture a full picture of people’s news consumption. A granular examination of website-tracking data to pinpoint what types of news coverage individuals frequently visit for coping with the pandemic will be informative. Last, future inquiries can compare the influence of negative portrayals of Asians circulated through traditional media and social networking sites on out-group attitudes and behavioral avoidance using experimental designs.

### Conclusions

Experiencing racial discrimination among the Asian population during a challenging pandemic could cause poor psychological outcomes and exacerbate health disparities [62]. The results suggest that conservative ideology, personal infection history, intergroup contact, traditional news exposure, and trust in social media positively associate with prejudice against Asians and Asian Americans. Conversely, relying on left-leaning and balanced news outlets is related to less prejudice. For those who have more trust in social media and digital news, frequent use of these two sources is associated with lower levels of prejudice. This research highlights the urgent need to reshape part of the traditional news discourses. Emphasizing scientific evidence and political institutions’ responsibility for creating effective solutions rather than placing the blame on ethnic minorities will decrease the citizens’ perceptions of infection threats, thereby reducing disease-related racial prejudice. It is also critical for public health organizations to leverage social media and mobile news apps for developing credible messages to combat racial discrimination against Asians linked to the COVID-19 pandemic.

## Abbreviations

H: hypothesis
MERS: Middle East respiratory syndrome
Mturk: Mechanical Turk
RQ: research question
